# Prolonged Voluntary Running Negatively Affects Survival and Disease Prognosis of Male SOD1G93A Low-Copy Transgenic Mice

**DOI:** 10.3389/fnbeh.2018.00275

**Published:** 2018-11-13

**Authors:** Luciana Garbugino, Elisabetta Golini, Alessandro Giuliani, Silvia Mandillo

**Affiliations:** ^1^Istituto di Biologia Cellulare e Neurobiologia, Consiglio Nazionale delle Ricerche, Rome, Italy; ^2^Environment and Health Department, Istituto Superiore di Sanità, Rome, Italy

**Keywords:** ALS, exercise, running wheels, sex differences, rotarod, Principal Component Analysis

## Abstract

Amyotrophic Lateral Sclerosis (ALS) is a disease in which physical activity plays a controversial role. Epidemiological studies indicate an association between intense exercise and risk of developing ALS. To study the impact of physical activity on ALS, mouse models rely mostly on forced exercise. In this study we hypothesized that voluntary wheel running could represent a better model of the influence of exercise in the pathogenesis of ALS. We used an automated home-cage running-wheel system that enables individual monitoring of performance. To verify the effect of voluntary running on disease progression, prognosis and survival as well as motor functions, we challenged SOD1G93A low-copy male and female mice on one (1 RW, at age 24 weeks) or multiple (3 RW) running sessions at age 13, 18, and 24 weeks. In parallel we measured performance on Rotarod and Grip strength tests at different ages. Several parameters were analyzed through Principal Component Analysis in order to detect what indices correlate and may be useful for deeper understanding of the relation between exercise and disease development. We found mutant male mice more negatively affected than females by prolonged and repeated exercise. SOD1G93A low-copy male mice showed shorter survival, increased body weight loss and poorer disease prognosis when exposed to multiple running sessions. These findings could encourage the investigation of the pathogenetic mechanisms underlying the supposedly increased risk to develop ALS in humans engaged in specific and intense exercise activities.

## Introduction

Amyotrophic lateral sclerosis (ALS) is a fatal, multi-systemic disease in which motor neurons degenerate relentlessly affecting the neuromuscular system with a rapid progression that ends in paralysis and death for respiratory failure. Beside known genetic mutations, which are discovered at an increasing rate in both familial and sporadic ALS forms (Ajroud-Driss and Siddique, 2015; Al-Chalabi et al., 2017), a number of environmental risk factors have been associated to the disease: exposure to toxins, pesticides, heavy metals, diet, smoking, anti-inflammatory drugs, doping, and strenuous exercise (Al-Chalabi and Hardiman, 2013; Oskarsson et al., 2015; Bozzoni et al., 2016). However, it is still controversial whether exercise has beneficial or detrimental effects on disease onset and progression. Understanding the impact of physical activity in ALS is thus crucial. It seems that in humans, excessive exercise would affect a predisposed genetic profile; recent studies indicate a predisposition toward an “athletic” phenotype that increases susceptibility to ALS (Huisman et al., 2013). While several animal studies rely on forced exercise, we propose that voluntary physical activity (i.e., wheel running) could represent a better tool to model the influence of environmental factors in the pathogenesis of ALS. When considered as a model of human leisure physical activity, voluntary running in mice also provides a reliable representation of individual variability. We hypothesize that prolonged voluntary running could be detrimental in predisposed individuals (i.e., SOD1 mutant mice) and worsen disease prognosis.

We investigated the effects of wheel running in a genetic mouse model of ALS, B6.Cg-Tg(SOD1^*^G93A)^dl^1Gur/J (SOD1G93A low-copy) expressing about 8–10 copies of the transgene, 30% fewer copies than the standard SOD1G93A model (Gurney et al., 1994). These mice show delayed onset and slower disease progression and develop paralysis between 28 and 34 weeks of age. This model, used in longitudinal studies, gives the opportunity to examine the very early stages of pathology and allows a longer therapeutic window to test new interventions (Alexander et al., 2004; Acevedo-Arozena et al., 2011). This strain has been already analyzed in our lab with an automated running wheel system and has shown motor deficits as early as 12 weeks of age (Mandillo et al., 2014).

The aim of this study was to verify the effect of voluntary wheel running on the disease onset, progression, motor function (muscular strength, motor coordination and running) and survival of male and female SOD1G93A low-copy mice by challenging them with one or multiple 3-weeks running sessions. We consider very important to focus on the analysis of both male and female individuals as in this and other models of neurodegeneration, the incidence of the disease is often different in the two sexes and very few animal studies tackle this issue (Clayton and Collins, 2014).

Several parameters including motor behavior were analyzed through Principal Component Analysis in order to detect what indices of the different tests may be correlated and useful to understand the impact of exercise on the development of the disease.

We expected wheel running activity, behavioral performance and survival to be differentially altered in male and female SOD1G93A low-copy mice depending on exposure to previous running sessions. In particular, mutant male mice exposed to 3 running wheel sessions showed increased body weight loss, shorter survival and poorer disease prognosis.

## Materials and methods

### Animals and husbandry

Adult male mice of B6.Cg-Tg(SOD1^*^G93A)^dl^1Gur/J (SOD1G93A low-copy) on a C57BL/6J background were initially obtained from MRC (Harwell, UK) and subsequently bred in-house at the CNR-EMMA facility (Monterotondo, Italy) by crossing hemizygous transgenic males with C57BL/6J females. Male and female mice obtained from three consecutive generations were employed in the study. Wild type littermates (WT) were used as controls. Mice transgenic for the human SOD1G93A mutation represent a widely used model of Amyotrophic Lateral Sclerosis (ALS). Compared to the most used B6.Cg-Tg(SOD1^*^G93A)1Gur/J transgenics (Gurney et al., 1994), which carry ~25 copies of the human transgene, SOD1G93A low-copy mice carry ~ 8–10 copies, and show a later onset of pathology and prolonged survival (Alexander et al., 2004; Acevedo-Arozena et al., 2011).

Sex and age matched littermates were group-housed, 3–5 per cage, in standard cages (Thoren, Hazleton, PA, USA) enriched with a transparent red polycarbonate igloo house (Datesand, Manchester, UK) and with wood shavings contained in single cellulose bags (Scobis Uno bags, Mucedola, Settimo Milanese, Italy). Food (2918 Teklad diet, Mucedola, Settimo Milanese, Italy) and water were available *ad libitum*. Room temperature was 21 ± 2°C, relative humidity was 50–60%, and mice were kept in a 12 h light/dark cycle with lights on at 8 am. Animals were subjected to experimental protocols approved by the Veterinary Dept. of the Italian Ministry of Health, and experiments were conducted according to the ethical and safety rules and guidelines for the use of animals in biomedical research provided by the relevant Italian laws and European Union's directives (Italian Legislative Decree 26/2014 and 2010/63/EU). All adequate measures were taken to minimize animal pain or discomfort.

### Experimental design and groups

**Figure 1:**
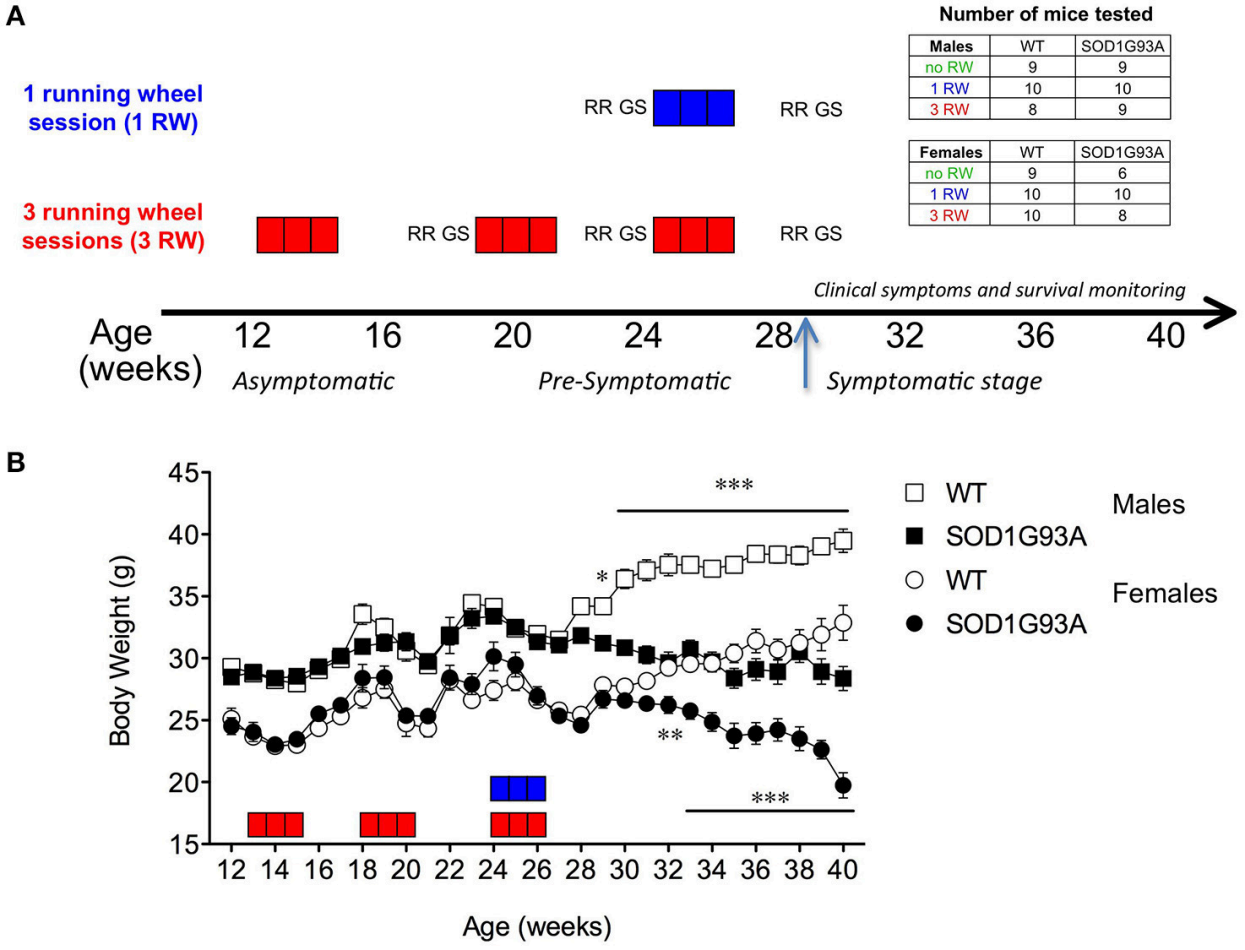
Experimental design and body weight. **(A)** Experimental design: Each square box represents 1 week of running wheel (RW) exposure: in blue, group 1 RW; in red, group 3 RW. Each three squares/weeks is one session. RR, Rotarod test; GS, grip strength test. Arrow indicates appearance of disease symptoms and beginning of health and survival daily monitoring. Inset: Tables indicate the number of mice tested in each experimental group. **(B)** Body weight curve in grams of male and female SOD1G93A low-copy and WT littermates across experimental window (12–40 weeks of age). Number of animals varied along the study. Age 12–18 weeks (3 RW only): *n* = 8–10 per sex/genotype; Age 19–30 weeks: *n* = 23–29 per sex/genotype; Age 30–40 weeks: WT *n* = 26–29 per sex, SOD1G93A *n* = 6–20 per sex depending on survival. Asterisks indicate statistically significant differences between WT and transgenic mice in each sex group. ^*^P < 0.05, ^**^P < 0.01, ^***^P < 0.001.

Mice were divided in three experimental groups for different exposure to exercise: (i) no exposure (no RW), (ii) 1 running session in which mice were exposed to one running wheel session at the age of 24 weeks (1 RW) and (iii) 3 running sessions in which mice were exposed to three running wheel sessions at the age of 13, 18, and 24 weeks (3 RW). Each running session lasted 3 weeks. Mice of the “no RW” group were left undisturbed in their home cages and used for comparison with the exercise exposed mice. Mice exposed to running wheels were also tested on rotarod and grip strength tests in between (3 RW group) or before and after (1 RW group) running wheel sessions (Figure 1A). The number of mice tested in each experimental group are reported in Figure 1A.

### Wheel running activity

During the running wheel sessions, mice were single-housed and a running wheel (TSE Systems, Bad Homburg, Germany) was placed in each home cage (Type II long, Ebeco, Germany) in a dedicated room with the same light/dark cycle and environmental conditions as in the housing room. Data of 3 consecutive weeks of wheel running activity were automatically collected and processed with PhenoMaster software (TSE Systems). In week 1 and week 2, mice were exposed to a standard wheel, while during week 3 a “complex” wheel was introduced. This wheel has a fewer number of rods which are irregularly spaced, though with a predictable pattern (Liebetanz and Merkler, 2006; Mandillo et al., 2014). Food and water were available *ad libitum*, and nestpaper was provided. Cages were changed every week. Wheel running activity for the 12 h dark phase was included in this study. Parameters measured were weekly values of: (1) total distance (m); (2) average run duration (s), where “runs” are running episodes at a velocity exceeding 30 rpm (~0.18 m/s); (3) average maximum speed (m/s) from daily values calculated as average of maximum speed values sampled every 5 min during the 12 h dark period; (4) total time on wheels (s).

### Rotarod

Mice of the 3 RW group were tested on the Rotarod at 17, 23, and 29 weeks of age, while mice of the 1 RW group were tested at 23 and 29 weeks (Figure 1A). Mice had to keep their balance on a rotating rod (3-cm diameter) set at an accelerating speed from 4 to 40 rpm in 300 s (mod. 47600 apparatus; Ugo Basile, Como, Italy). To familiarize with the apparatus mice underwent a training session of 3 trials, 60 s each, in which the rod was kept stationary for the first trial and held at 4 rpm for the last two trials. The next day, for 4 consecutive days, mice were tested over 3 trials/day with an inter-trial interval of ~30 min. A maximum of three mice were placed on the rod at the same time. The latency to fall from the rotating rod was recorded in each trial. If a mouse was passively rotating on the rod (i.e., clinging) the number of passive rotations were counted. For each day data were expressed as mean latency to fall minus 1 s “penalty” for each passive rotation (Marazziti et al., 2013). Mean 4-days latency was considered for analysis.

### Grip strength

Mice of the 3 RW group were tested on the grip strength meter apparatus (Bioseb, France) at age 18, 24, and 30 weeks, while mice of the 1 RW group were tested at 24 and 30 weeks (Figure 1A). The mouse was held gently by the base of its tail over the top of the grid enabling forelimbs and hindlimbs to grip the grid. With its torso in a horizontal position the mouse was pulled back steadily until the grip was released down the complete length of the grid. The propensity is that the mouse will cling onto the grid until it can no longer resist the increasing force, before it is released (Mandillo et al., 2008). The grip strength meter digitally displays the maximum force applied as the peak tension (in grams) once the grasp is released. The mean of five consecutive trials was taken as an index of forelimb and hindlimb grip strength. Mice were given an inter-trial interval of about 60 s. Body weight was taken at the end for further analyses.

### Body weight, symptoms, and humane endpoint assessment

Body weight (BW) was measured weekly from age 12 weeks (Figure 1B). From age 30 weeks, daily monitoring of disease symptoms was performed: tremors, hind limb extension reflex loss, abnormal gait and posture, bradykinesia, and paralysis were annotated.

Extra wet food was provided as needed. Humane endpoint was set at limb paralysis onset or when weight drop exceeded 25% from peak body weight. Disease onset was defined as appearance of symptoms and the time at which the mouse permanently starts to lose weight (age at peak body weight). Mice that reached humane endpoint were sacrificed by carbon dioxide inhalation.

### Statistical analysis

A repeated-measures ANOVA (RM ANOVA) was performed on most datasets with Genotype, Sex and Exercise as between-subject factors and Age or Time as within-subject factors. *Post-hoc* analysis was performed where possible.

Three independent principal component analyses (PCA) (Giuliani, 2017) were performed including all animals (total *n* = 108), only running mice (*n* = 75), and only mutant mice (*n* = 52), respectively. In turn, for each of the above groups, PCA was applied separately for three groups of variables, namely: (1) Body weight, rotarod latency, grip strength; (2) wheel-running distance and run duration, time on wheels; (3) age (days) at peak BW (disease onset), survival and duration of disease (survival days minus disease onset).

Pearson's correlations and three-way ANOVAs with Genotype, Sex and Exercise as main factors were then separately performed on the extracted components. Unpaired *t*-tests and Chi-square tests were also performed to check specific hypotheses.

Subjects whose individual values differed more than 2 standard deviation units in module from the group average were considered outliers and were excluded from analysis. Significance level was set at *P* < 0.05. Data are presented as mean ± s.e.m. All statistics were run using the StatView 5.0 PowerPC, SAS System 9.1 (SAS Institute Inc., Cary, NC) and Prism 5.0a (GraphPad Software Inc., La Jolla, CA, USA) software packages.

## Results

### Exercise induces significant body weight changes in male SOD1G93A low-copy mice

Figure 1B shows the time course of body weight across the experimental window, from 12 to 40 weeks of age in male and female SOD1G93A low-copy mice and their WT littermates. SOD1G93A low-copy mice showed significantly lower body weight compared to WT starting from 29 weeks of age in males (*P* < 0.05) and from 32 weeks of age in females (*P* < 0.01), suggesting a delayed disease onset in female mice.

**Figure 2:**
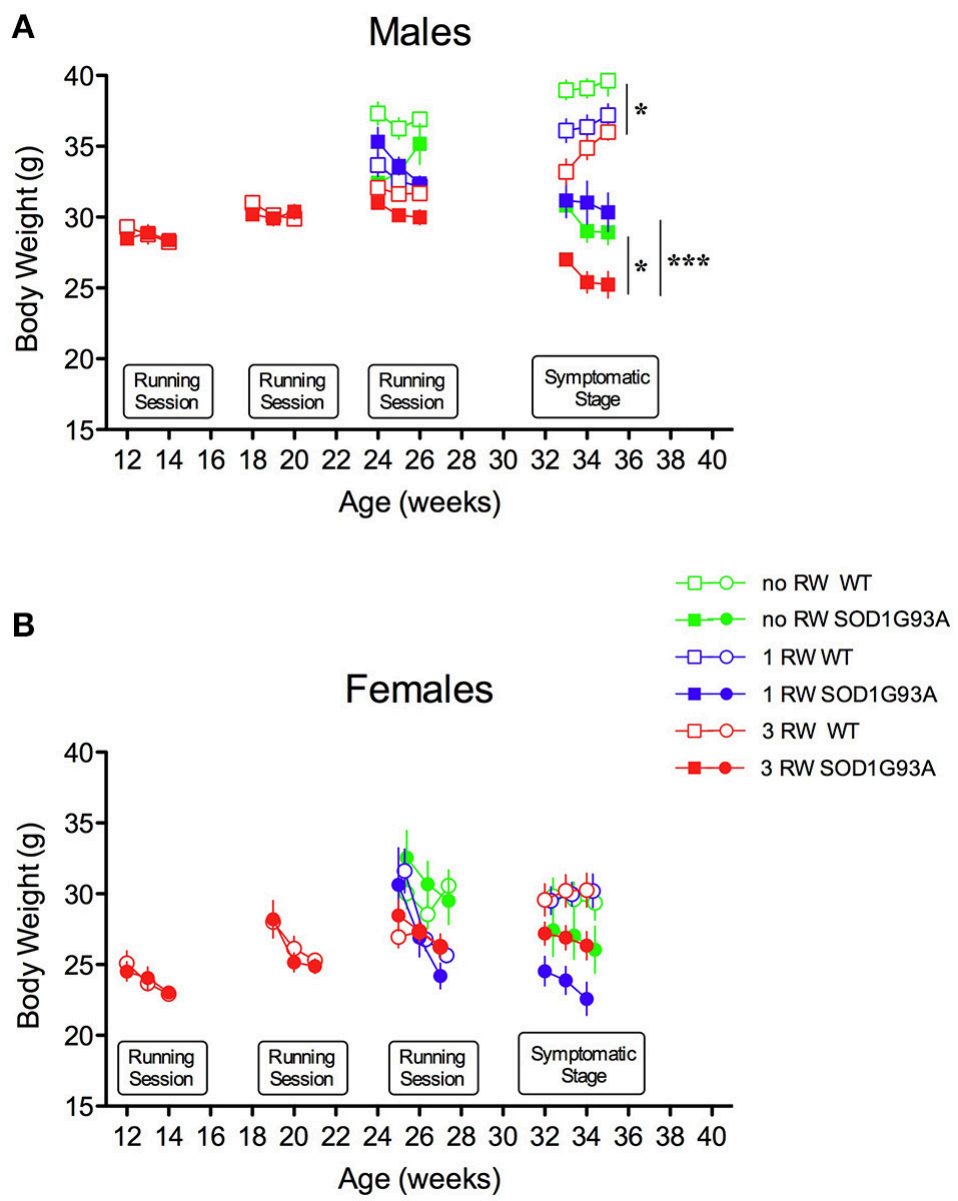
Effect of running wheel exercise on body weight. **(A)** Effect of exercise on body weight of male mice during running sessions and at symptomatic stage. ANOVA, Running session at 24–26 weeks: Genotype effect, *F*_(1, 47)_ = 4.69, *P* = 0.035; Exercise effect, *F*_(2, 47)_ = 12.53, *P* < 0.0001, Genotype × Exercise, *F*_(2, 47)_ = 4.853, *P* = 0.012. ANOVA, Symptomatic stage (age 32–34 weeks): Genotype effect *F*_(1, 49)_ = 120.3, *P* < 0.0001; Exercise effect *F*_(2, 49)_ = 10.74, *P* = 0.0001. *n* = 8–10 per group. ^*^P < 0.05 no RW vs. 3 RW in WT and SOD1G93A low-copy mice, ^***^P < 0.001 1 RW vs. 3 RW in SOD1G93A low-copy mice, Bonferroni *post-hoc* test. **(B)** Effect of exercise on body weight of female mice during running sessions and at symptomatic stage. ANOVA, Running session at 24–26 weeks: Exercise effect, *F*_(2, 43)_ = 4.446, *P* = 0.017. ANOVA, Symptomatic stage (age 32–34 weeks): Genotype effect, *F*_(1, 43)_ = 17.18, *P* = 0.0002. *n* = 6–10 per group.

Exercise exposure affected body weight in a gender and genotype-dependent manner (Figure 2). Male SOD1G93A low-copy mice exposed to 3 sessions of running activity showed a lower body weight compared to mice of the same genotype that had only one session of running or no running exposure (Figure 2A). This difference due to exercise exposure further increased in SOD1G93A with the progression of the disease when observed at the symptomatic stage. On the other hand, body weight of female SOD1G93A low-copy mice did not show overt decrease after prolonged exercise exposure, compared to 1 RW (Figure 2B). It appeared that repeated exercise sessions (3 RW group) canceled sex differences in body weight of transgenic mice (male and female mice weighted, respectively, g 26.03 (±0.46) and g 26.81 (±0.86) at the symptomatic stage). As expected, mice not exposed to running wheels weighed more than exercised mice. This difference was more pronounced in males.

### Repeated exercise reduces survival of male SOD1G93A low-copy mice

**Figure 3:**
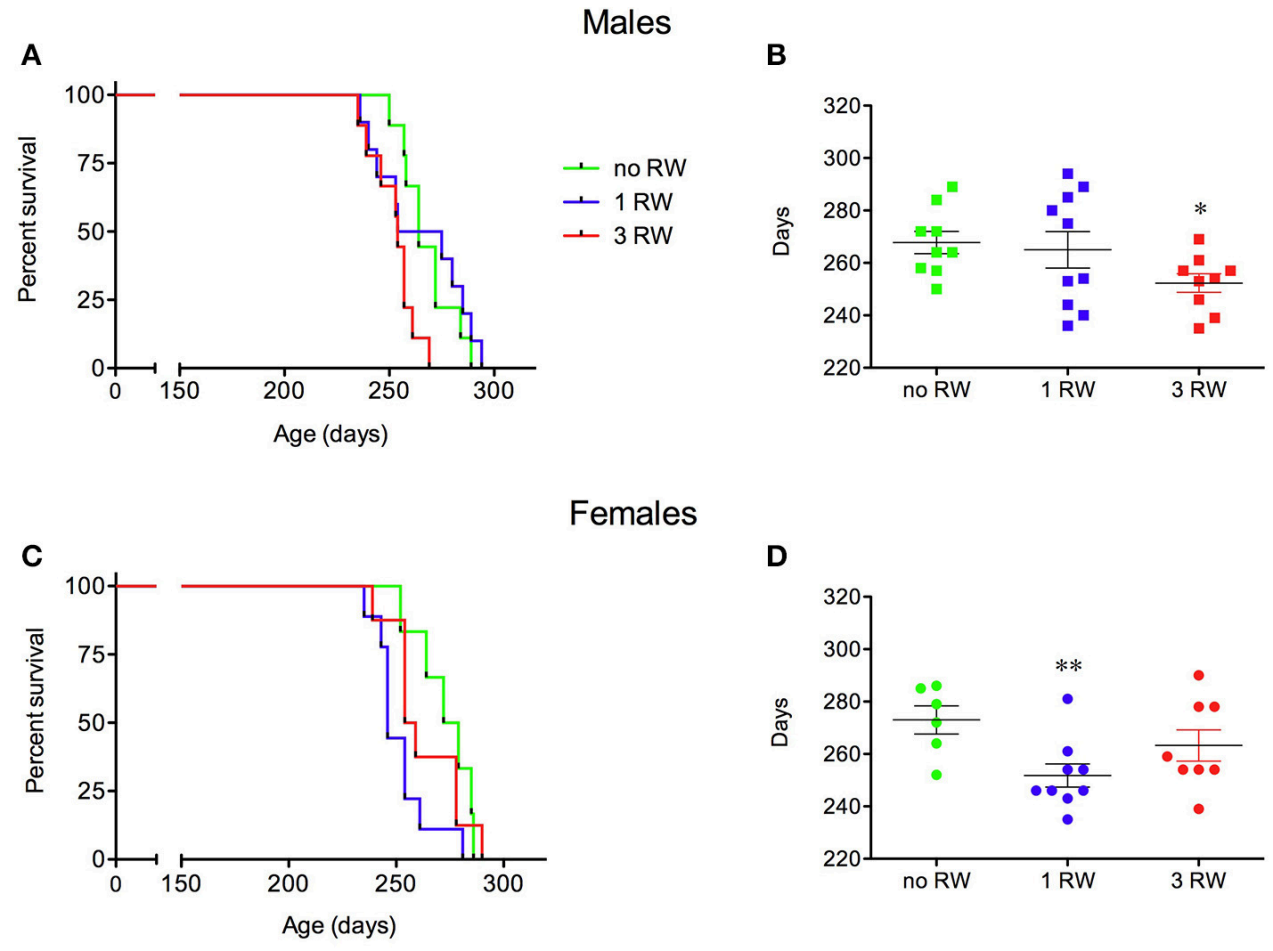
Effect of exercise experience on survival of SOD1G93A low-copy mice. **(A–C)** Kaplan-Meier survival curves for male **(A)** and female **(C)** mice from the exercise groups (1 and 3 RW) and from a group never exposed to running wheel exercise (no RW). **(B–D)** Scatter plot of survival days (mean ± s.e.m.) for male **(B)** and female **(D)** mice from the three experimental groups. ^*^P < 0.05, no RW vs. 3 RW, *t*-test; ^**^P < 0.01, no RW vs. 1 RW, *t*-test. *n* = 6–10 per group.

Different levels of exercise also affected survival of SOD1G93A low-copy mice in a sex-dependent fashion (Figure 3). Males exposed to higher level of exercise (3 RW) and females exposed to only one session (1 RW) had a shorter survival than animals never exposed to a running wheel (Figure 3A, Log-rank test *P* = 0.0075; Figure 3C, Log-rank test *P* = 0.02).

In particular, male SOD1G93A low-copy mice of the 3 RW group had a shorter life span (252.3 ± 4 days) than the animals exposed to only one session of running activity (1 RW, 265 ± 7 days) or mice that were never exposed to the running wheel (no RW, 267.8 ± 4 days) (Figure 3B). Females (Figure 3D) had a shorter life span when exposed to 1 RW session (251.8 ± 4 days) compared to the no RW group (273 ± 5 days) or the 3 RW (263.3 ± 6 days).

### Previous running experience improves male SOD1G93A mice running performance

**Figure 4:**
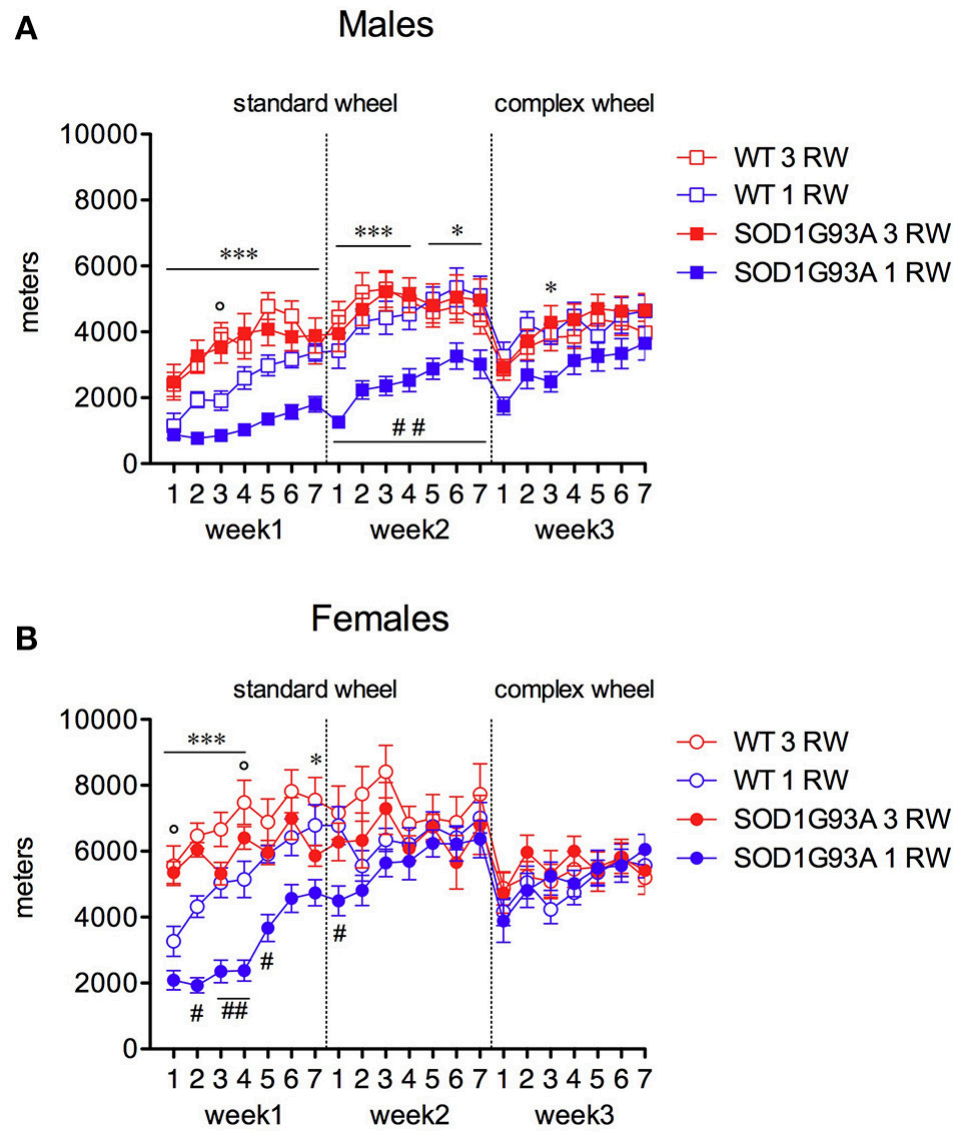
Effect of exercise experience on running distance at 24–26 weeks of age. Total distance traveled (meters) on standard and complex running wheels in male and female mice at 24–26 weeks (nightly running mean ± s.e.m. across three-week's session). **(A)** Males, RM ANOVA: Genotype factor, *F*_(1, 33)_ = 4.584, *P* = 0.04; Exercise factor, *F*_(1, 33)_ = 12.695, *P* = 0.001; Genotype × Exercise, *F*_(1, 33)_ = 6.011, *P* = 0.02. **(B)** Females, RM ANOVA: Genotype factor, *F*_(1, 34)_ = 3.553, *P* = 0.06; Exercise factor, *F*_(1, 34)_ = 8.789, *P* = 0.0055; Genotype × Exercise, n.s. Bonferroni *post-hoc*'s: ^***^P < 0.001, ^*^P < 0.05 1 RW vs. 3 RW in SOD1G93A low-copy mice; °*P* < 0.05 1 RW vs. 3 RW in WT mice; ^#^*P* < 0.05; ^##^*P* < 0.01 WT vs. SOD1G93A low-copy in 1 RW group. *n* = 8–10 per group.

**Figure 5:**
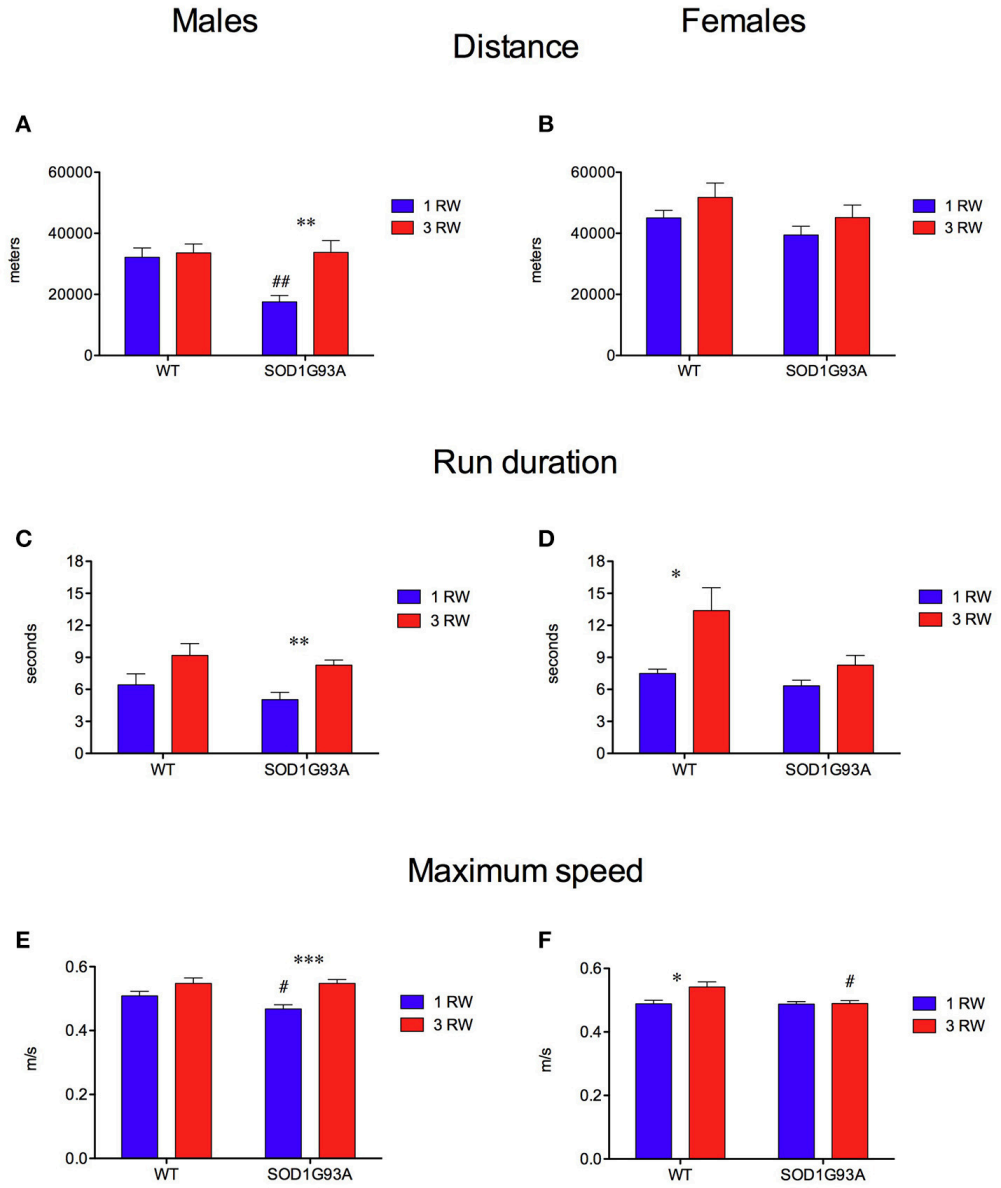
Effect of exercise experience on running parameters during week 2 at age 24–26 weeks. **(A,B)** Total distance traveled (meters) on running wheels in male and female mice (mean±s.e.m. of session's second week). **(A)** Males, ANOVA: Genotype factor, *F*_(1, 33)_ = 5.674, *P* = 0.023; Exercise factor, *F*_(1, 33)_ = 8.502, *P* = 0.0063; Genotype × Exercise, *F*_(1, 33)_ = 5.906, *P* = 0.02. *t*-test: ^**^P = 0.0014 1 RW vs. 3 RW in SOD1G93A low-copy mice; ##*P* = 0.001 WT vs. SOD1G93A low-copy in 1 RW group. **(B)** Females, ANOVA: All factors are n.s. **(C,D)** Mean duration (seconds) of running episodes (>30 rpm) on running wheels in male and female mice (mean ± s.e.m. of session's second week). **(C)** Males, ANOVA: Exercise factor, *F*_(1, 33)_ = 12.179, *P* = 0.0014, other factors are n.s. *t*-test: ^**^P = 0.0014 1 RW vs. 3 RW in SOD1G93A low copy mice. **(D)** Females, ANOVA: Genotype factor, *F*_(1, 34)_ = 6.259, *P* = 0.017; Exercise factor, *F*_(1, 34)_ = 9.774, *P* = 0.0036. ^*^P = 0.014 1 RW vs. 3 RW in WT mice. **(E,F)** Average maximum speed (m/sec) of running in male and female mice (mean ± s.e.m. of session's second week). **(E)** Males, ANOVA: Exercise factor, *F*_(1, 33)_ = 18.311, *P* = 0.0002; *t*-test: ^***^P = 0.0003 1 RW vs. 3 RW in SOD1G93A low-copy mice; ^#^*P* = 0.04 WT vs. SOD1G93A low-copy in 1 RW group. **(F)** Females, ANOVA: Genotype factor, *F*_(1, 34)_ = 5.022, *P* = 0.03; Exercise factor, *F*_(1, 34)_ = 5.226, *P* = 0.028; Genotype × Exercise, *F*_(1, 34)_ = 4.499, *P* = 0.04. *t*-test: ^*^P = 0.016 1 RW vs. 3 RW in WT mice; ^#^*P* = 0.02 WT vs. SOD1G93A low-copy in 3 RW group. *n* = 8–10 per group.

Exercise experience also affected running performance in a genotype and sex-dependent manner (Figures 4, 5). Running wheel activity was compared between the 1 RW and 3 RW groups at the age of 24–26 weeks. Previous exercise experience (3 RW) seemed to bring running performance of transgenic mice to levels comparable to WT in both males and females. Performance on the complex wheel during the third week was reduced similarly in all groups (Figure 4). In particular, running performance (distance, run duration and speed) was analyzed during the second week of the session as in a previous study (Mandillo et al., 2014), we found that animals improved their performance throughout the first week of wheel running, whereas this reached a stable plateau and remained consistent over the second week (Figure 4, week2 and Figure 5). SOD1G93A low-copy male mice of the 3 RW group compared to the 1 RW group, showed higher levels of distance traveled (Figures 4, 5A), run duration (Figure 5C) and maximum speed reached (Figure 5E). No such differences were observed in WT males (Figures 5A,C,E). Overall, female mice ran more (Figure 4) and for longer bouts than males [Figures 5B,D; ANOVA Sex factor: Distance *F*_(1, 67)_ = 46.029, *P* < 0.0001; Run Duration *F*_(1, 67)_ = 4.592, *P* < 0.05] but both sexes showed similar running speeds (Figures 5E,F).

### Rotarod and grip strength performance is affected by exercise experience

**Figure 6:**
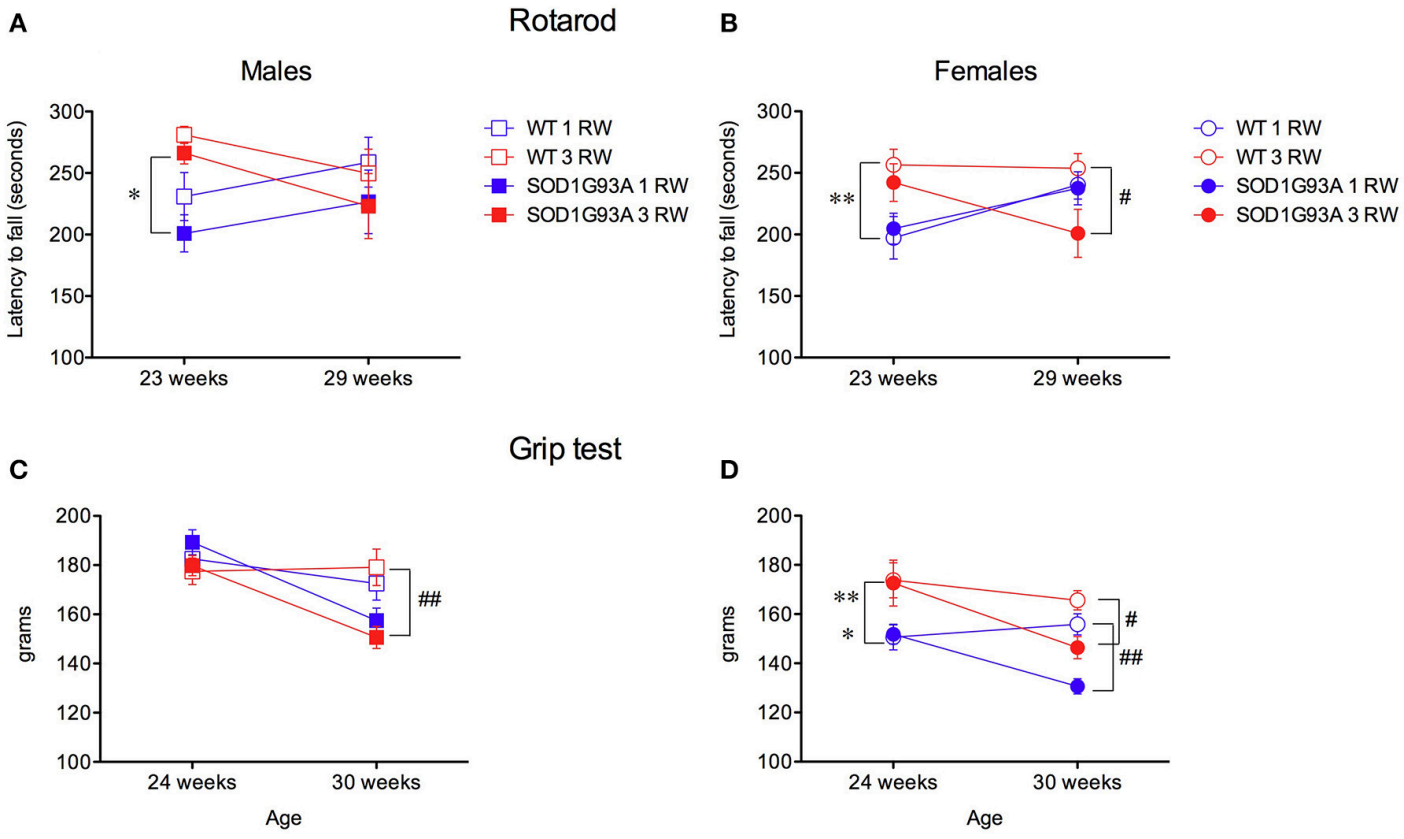
Effect of exercise experience on Rotarod and Grip strength tests. **(A,B)** Rotarod test. Latency to fall (seconds, mean±s.e.m. of 3 trials/4 days) from rod in male and female mice at age 23 and 29 weeks. Bonferroni *post-hoc* tests: ^*^P < 0.05, ^**^P < 0.01 1 RW vs. 3 RW; ^#^*P* < 0.05 WT vs. SOD1G93A. **(C–D)** Grip strength test (grams, 4 paws mean ± s.e.m. of 5 trials) in male and female mice at age 24 and 30 weeks. Bonferroni *post-hoc* tests: ^*^P < 0.05 1 RW vs. 3 RW in SOD1G93A, ^**^P < 0.01 1 RW vs. 3 RW in WT; ^#^*P* < 0.05 WT vs. SOD1G93A in 3 RW, ^##^*P* < 0.01 WT vs. SOD1G93A in 3 RW males and in 1 RW females.

In the rotarod test, animals of the 3 RW group showed longer latencies to fall compared to the 1 RW group in both males and females at 23 weeks of age [Figures 6A,B; ANOVA Exercise factor: *F*_(1, 67)_ = 26.621, *P* < 0.0001]. At that age (23 weeks) mice of the 3 RW and 1 RW groups had run for 2 or none RW sessions, respectively. Conversely, no effect of exercise was observed on the rotarod at 29 weeks of age but at this time point transgenic mice performed worse than WT [Figures 6A,B; ANOVA Genotype factor, *F*_(1, 67)_ = 4.398, *P* = 0.03]. A statistically significant interaction between Age and Exercise was in fact found in both sexes [RM ANOVA, *F*_(1, 67)_ = 24.622, *P* < 0.0001].

Grip strength performance was positively affected by repeated exercise only in females [Figure 6D; RM ANOVA, Sex × Exercise group *F*_(1, 67)_ = 15.344, *P* = 0.0002]. As expected, transgenic SOD1G93A low-copy mice showed a significant decline in grip strength at the age of 30 weeks [Figures 6C,D; ANOVA, *F*_(1, 67)_ = 37.722, *P* < 0.0001].

### Repeated exercise worsen disease prognosis in male SOD1G93A mice

To have a comprehensive view of the results described so far, a Principal Component Analysis (PCA) was performed on a selection of variables from the various tests and measures. A first analysis was conducted on all mice (both running and non-running) by considering the following variables: body weight at age 22–24 weeks (pre-symptomatic stage) and 32–34 weeks (symptomatic stage), rotarod latency to fall at age 23 and 29 weeks, grip strength at age 24 and 30 weeks. From the PCA, three components (factors) emerged accounting for 78.2% of the total variance: on scree-test inspection (Giuliani, 2017) this three component solution can be considered a reliable global representation of the data set. Table 1 illustrates the component loadings pattern (loadings correspond to the Pearson correlation between original variables and components). The first Principal Component (PC1) accounted for 35.8% of the variance, it is a “size” component (Jolicoeur and Mosimann, 1960) with all positive loadings for the variables related to physical strength. PC1 positively correlates with both rotarod and grip tests and can thus be interpreted as the “force” related to body mass. The second component (PC2) accounted for 29% of the variance and gives a different perspective on the effect of physical strength (indicated by body weight with high and positive loadings) on rotarod performance (with negative loadings). This inverse relation between body weight and rotarod performance can be interpreted as “motor coordination or agility” that, as expected, has no relation with grip strength performance (low loadings). In other words the same measure (body weight) exerts two opposite (and mutually independent) effects on rotarod performance: a positive one (PC1) related to the increase in physical strength and a negative one (PC2) related to the decrease in agility.

**Table 1:** Principal component analysis on all mice data.

	**Components (variance explained)**		
**Variables**	**PC1 (35.8%)**	**PC2 (29%)**	**PC3 (13.4%)**
Body Weight 22–24 weeks	0.400	**0.796**	0.012
Body Weight 32–34 weeks	**0.684**	0.494	−0.354
Rotarod latency 23 weeks	0.543	–**0.675**	0.140
Rotarod latency 29 weeks	**0.605**	–**0.582**	−0.114
Grip strength 24 weeks	**0.581**	0.221	**0.753**
Grip strength 30 weeks	**0.722**	−0.092	−0.287

*Body weight, rotarod and grip test variables from all mice tested (n = 108). A three components solution was obtained, accounting for 78.2% of total variance. PC1 was interpreted as “force” related to body mass, PC2 as “coordination or agility” unrelated to strength and PC3 as a singularity of the grip strength at age 24 weeks. Data are factor loadings (in bold ≥ |0.6|)*.

The third component (PC3) accounted for 13.4% of the variance and is associated to a singularity of the grip test only at 24 weeks not easy to interpret.

When further analyzing by ANOVA the two PCs (PC1 = “force”; PC2 = “coordination”), we found statistically significant effects as for Genotype, Sex, Exercise, and Sex × Exercise for the component “force” (Supplementary Table 1). For the “coordination” component, a statistically significant effect was found only for the factors Sex and Exercise but not for Genotype.

**Figure 7:**
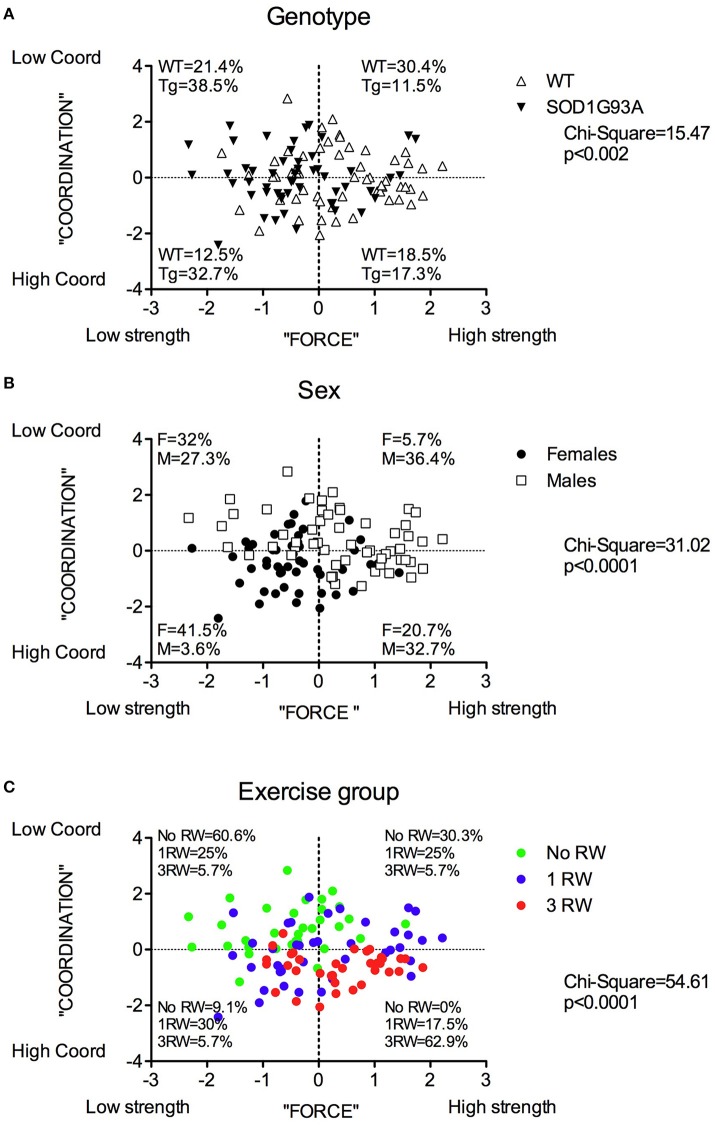
Distribution of mice in the PC1 “Force” and PC2 “Coordination” principal components space for each between subjects factor: Genotype, Sex and Group. Distribution and percentage of individuals of **(A)** the two genotypes, **(B)** the two sexes, **(C)** the three exercise groups in the four quadrants subdividing the “Force”/”Coordination” space. Chi-square values are significant for each factor.

The distribution of the animals in the PC1/PC2 (strength/coordination) plane in terms of their experimental groups is reported in Figure 7. SOD1G93A individuals are mostly distributed in the “low strength” quadrants (Figure 7A). Similarly, more females than males fall in the “low strength” quadrant (Figure 7B). As somewhat expected, individuals exposed to repeated exercise are mostly distributed in the “high strength/high coordination” quadrants, while the majority of no running mice fall in the “low strength/low coordination” quadrants (Figure 7C).

**Figure 8:**
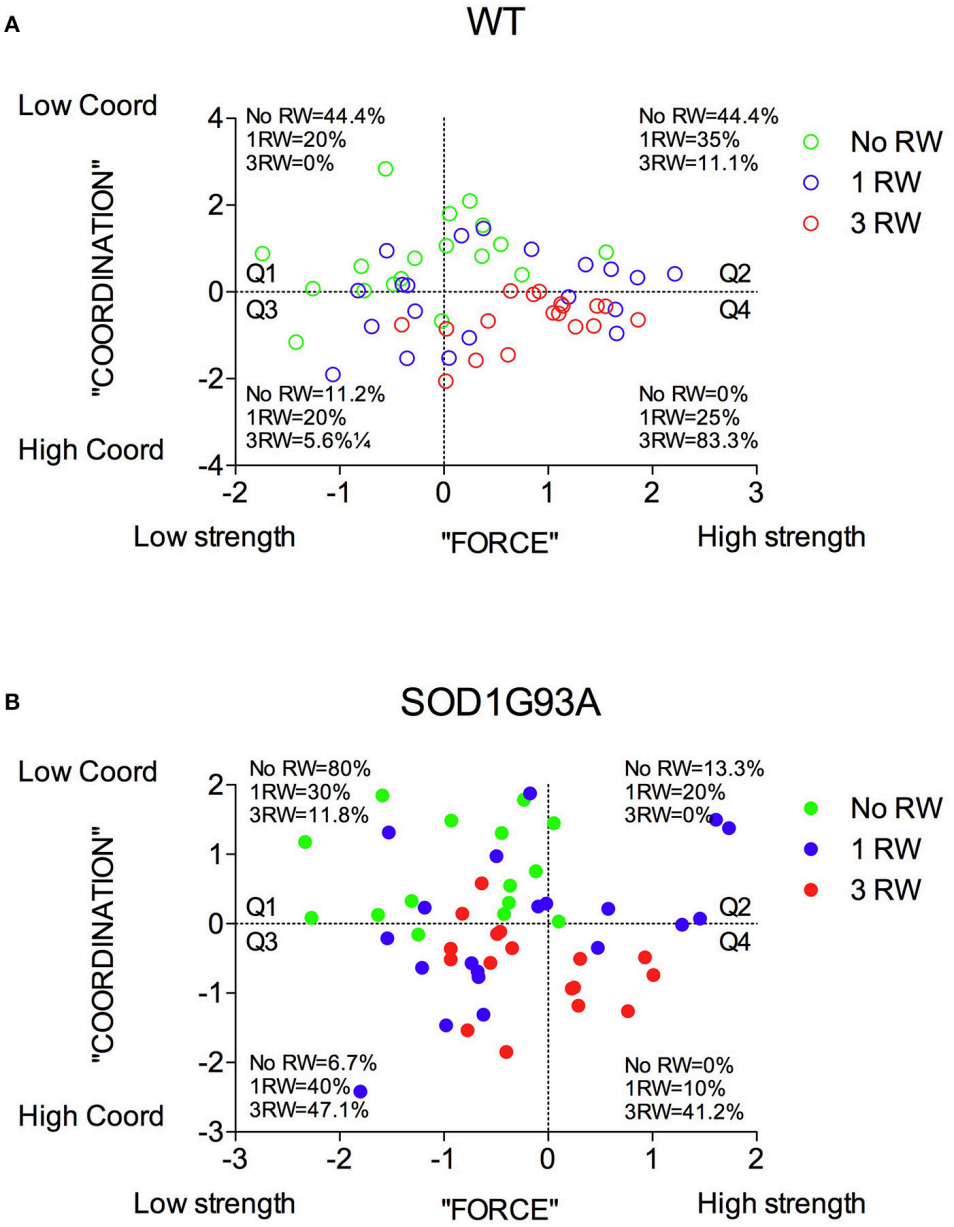
Distribution of mice from the three exercise groups in the PC1 “Force” and PC2 “Coordination” principal components space. **(A)** Wild-type and **(B)** SOD1G93A mice distribution and percentage of individuals of the three exercise groups in the four quadrants subdividing the “Force”/“Coordination” space.

In Figure 8 are depicted the distributions of WT and SOD1G93A low-copy mice of the three groups (No RW, 1 RW, 3 RW) in the PC1 and PC2 principal components space and the percentage of individuals of each group that occupy each quadrant. In particular, WT mice exposed to 3 RW exercise sessions are found mainly (83%) on Q4 (High strength-High Coordination) quadrant, compared to only 41% of the 3 RW mutant mice. Regardless of exercise exposure, most of the mutant mice occupy Q1 and Q3 (Low strength) quadrants while the WT are found mainly in the Q2 and Q4 (High strength) quadrants. Additionally, mutant mice of the No RW group fall mainly (80%) in the Q1 (Low strength-Low Coordination) quadrant. Chi-square comparisons among all groups are statistically significant (Chi-square: WT = 31.96, *P* < 0.0001; SOD1G93A = 26.81, *P* = 0.0002).

We then analyzed only the animals that had access to the running wheel for one (1 RW) or three sessions (3 RW). The PCA performed on the running wheel variables (distance, run duration and time on wheels) revealed only one component that accounted for 69% of the variance and it was interpreted as “running wheel performance” (with the three factor loadings all positive and >0.77). The ANOVA on this component showed statistically significant effects of Genotype, Sex, and Exercise (Supplementary Table 1).

Finally, we focused only on the SOD1G93A low-copy mice by considering the parameters related to the disease: survival, days at peak body weight (i.e., disease onset) and duration of disease. From the PCA, two components clearly emerged, with the first accounting for the 67% and the second for 33% of variance. The first component is all due to the duration of the disease (Table 2) while the second is completely independent from the duration but is related to onset and survival (both high and positive loadings). The first can be interpreted as “duration” of the disease or with its “prognosis” while the second is related to the “time with no disease.” Oversimplifying we could name the first as “disease-related” and the second as “life time without disease.”

**Table 2:** Principal component analysis on SOD1G93A mice disease-related data.

	**Components (variance explained)**	
**Variables**	**PC1 (67%)**	**PC2 (33%)**
Age at peak BW (onset)	**−0.617**	**0.787**
Disease Duration	**0.999**	0.003
Survival	**0.795**	**0.606**

*Days at peak body weight (BW), i.e. age at disease onset, disease duration and survival variables from SOD1G93A low-copy mutant mice (n = 52). A clearly bipartite two components solution was obtained, accounting for 100% of total variance. PC1 was interpreted as “disease duration/prognosis,” PC2 as “life time without disease” unrelated to duration. Data are factor loadings (in bold >|0.6|)*.

**Figure 9:**
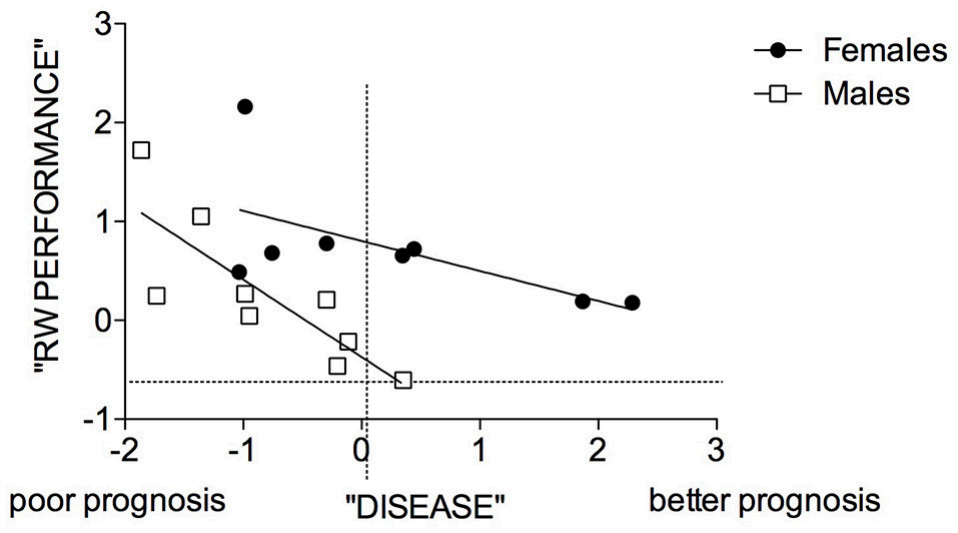
Correlation between “disease” and “RW performance” components in male and female SOD1G93A mice of the 3 RW exercise group. Symbols represent male and female mutant individuals exposed to 3 RW exercise sessions. Regression lines intercepts are significantly different [*F*_(1, 14)_ = 10.68, *p* = 0.0056].

Interestingly, the ANOVA on these 2 components revealed statistically significant effects of Exercise and Sex × Exercise interaction only for the “disease-related” component (Supplementary Table 1), no statistically significant effects were found for the second component. Correlating the component of “running wheel performance” with the “disease-related” component in the repeated exercise group (Figure 9) we found that in mutant males, the higher the RW performance the poorer the prognosis (Males: *r*^2^ = 0.68, *P* = 0.0065; Females: *r*^2^ = 0.38, *P* = 0.1) and that disease prognosis was worse in mutant males than females.

## Discussion

In our study we found that prolonged exercise exposure had a negative impact predominantly in male SOD1G93A low-copy mice. Namely, repeated wheel running reduced survival, exacerbated body weight decline and aggravated disease prognosis.

Even if exercise had deleterious effects on body weight and survival of transgenic male mice, previous running experience had, as expected, a positive effect on both running (Figure 4A) and rotarod performance (Figure 6A) in a similar way in the two genotypes, possibly indicating a training-induced phenomenon. In fact, beside the well-known positive influence of exercise on cognition reported in both animal and human studies (Hötting and Röder, 2013), also rotarod performance has been found improved in rats after prolonged voluntary wheel running due to better motor skill learning (Buitrago et al., 2004). In transgenic female mice repeated exercise induced a poor performance in the rotarod at 29 weeks while grip strength seemed affected positively by previous exercise experience at 24 weeks only in females (Figures 6C,D). Indeed we observed that the effects of voluntary exercise vary widely depending on the type of motor function or pathological stage. In fact, wheel running, rotarod and grip strength tests, measure different aspects of motor function (Mandillo et al., 2014) and they are not necessarily affected in the same way in the two sexes and in their interaction with the disease. The use of PCA allowed us to appreciate and interpret the impact of exercise exposure on different outcomes as body weight, motor performance, and disease progression to finally discriminate their relative contribution in a pathological condition as ALS in the two sexes. Most importantly it takes in to account the large individual variability observed in this mouse model. Also in humans, different types of exercise may have different impact on the health of individuals depending on their sex, genetic predisposition, life style, and past experiences (Hawley et al., 2014; Rosenfeld, 2017).

Some epidemiological evidences indicate a higher risk to develop ALS associated to intense exercise or professional sports, e.g., soccer, American football, baseball (Veldink et al., 2005; Beghi et al., 2010; Beghi, 2013). However, reports on the effects of exercise in ALS patients are rather controversial (Lisle and Tennison, 2015; Lacorte et al., 2016). Part of this dispute is due to non-standardized exercise protocols and paucity of large-scale human studies. In animal studies investigating the effects of exercise on ALS, very different protocols are used in terms of exercise type and duration. Most of them employ forced exercise protocols (i.e., treadmill and forced swim) that also induce a substantial amount of stress in the animal and are not so suitable to mimic human activities. We propose that voluntary exercise as the home cage wheel running used in our study, is a better model of human leisure physical activity.

While some studies found beneficial or no effect of moderate exercise on running wheels (Liebetanz et al., 2004; Kaspar et al., 2005; Bennett et al., 2014) only a few reported detrimental effects of intense forced exercise using the treadmill (Mahoney et al., 2004; Carreras et al., 2010).

Our study is the first demonstrating deleterious effects of voluntary prolonged running wheel activity. Moreover, we demonstrated that higher running wheel performance significantly correlates with poor prognosis only in mutant males. As shown in the PCA, exercise is somewhat beneficial in general but can be detrimental if associated to a disease related mutation. In humans, a genetic predisposition to physical fitness could be associated with higher risk of ALS (Mattsson et al., 2012; Huisman et al., 2013). Although proof of the association between physical activity and risk of ALS is still under debate (Lacorte et al., 2016), a recent survey demonstrated a positive association between sporadic ALS and long-term physical activity participation (Harwood et al., 2016).

We also showed clear sex differences in the impact of voluntary exercise on disease outcome with SOD1G93A females being less affected by prolonged physical activity but showing decreased survival after moderate exercise (1 RW) (Figures 3C,D). This could be due to their exposure to exercise for the first time at an age (24–26 weeks) that corresponds in this group to a critical phase of symptom onset, i.e., associated with a higher body weight drop (−20.9%) compared to 3 RW (−7.5%) and to males (1 RW: −8.3%; 3 RW: −3.5%). Nevertheless, females showed higher running activity than males but this did not seem to correlate with disease prognosis (Figure 9). A few other studies evaluated the effect of exercise on SOD1G93A male and female mice. They found that moderate treadmill forced exercise was beneficial only in males (Kirkinezos et al., 2003), only in females (Veldink et al., 2003) or, similarly to our findings, high intensity exercise was detrimental only in males (Mahoney et al., 2004). Moreover, we showed that at symptomatic stage mutant males lost more body weight than females after prolonged exercise, possibly due to sex differences in muscle metabolism. In fact, an additional explanation for the negative impact of this type of exercise in ALS could be due to unbalanced energy metabolism (Dupuis et al., 2011; Ferri and Coccurello, 2017; Vandoorne et al., 2018).

Controversial findings in both human and mouse studies could be in part attributed to poor quality data or at least excessive generalization on what is defined as exercise (Nikolaidis et al., 2012; Lisle and Tennison, 2015). Different types of exercise activities should also be considered in terms of their muscle-damaging impact. For example, repeated sprints and prolonged running as opposed to cycling and horizontal running are more prone to induce muscle damage via extended alteration of redox homeostasis (Nikolaidis et al., 2012). Voluntary wheel running in mice is a continuous repetition of running bouts. The parameter Running Performance used in the PCA of this study that is inversely correlating with disease prognosis, is the combination of running distance, running bout duration and time on wheel, thus reproducing the characteristics of human repeated sprints and prolonged running exercise. On the other hand, running on a complex wheel: 1) it requires different motor skills that are not related to general locomotion and 2) it implies shorter running bouts and reduced performance (Mandillo et al., 2014). In the present study, the use of the complex wheel had minimal impact on the relation between prolonged exercise and disease prognosis.

To contribute to the understanding of the mechanisms underlying the detrimental effects of voluntary prolonged and repeated exercise on several disease aspects (onset, progression, etiology, peripheral degeneration, neuromuscular pathology, inflammation and metabolism) further studies are in progress using SOD1G93A low-copy mice with the ambitious aim of proposing possible therapeutic interventions combining life style (exercise and diet) and new drug treatments to alleviate ALS symptoms. In conclusion our findings support the current guidelines on exercise interventions in ALS in which it is recommended the use of moderate rather than vigorous exercise activities (Majmudar et al., 2014). In addition, a personalized approach should be taken in ALS treatment according to genetic profile, disease stage and gender.

## Author contributions

LG and SM conceived and designed the experiments. LG performed the experiments. LG, EG, AG, and SM analyzed the data. EG and SM wrote the paper.

### Conflict of interest statement

The authors declare that the research was conducted in the absence of any commercial or financial relationships that could be construed as a potential conflict of interest. The handling editor declared a shared affiliation, though no other collaboration, with one of the authors AG at the time of the review.
