# Supplementary material for: Prolonged Voluntary Running Negatively Affects Survival and Disease Prognosis of Male SOD1G93A Low-Copy Transgenic Mice
Source: Front Behav Neurosci. 2018 Nov 13;12:275. doi: 10.3389/fnbeh.2018.00275 (PMC6243076; doi:10.3389/fnbeh.2018.00275)
Supplement: Supplementary file 1 [file Table_1.doc]

Suppl. Table 1. ANOVA on the most relevant Principal Components extracted from the PCA.

|  | *Components* | | | |
| --- | --- | --- | --- | --- |
| *ANOVA FACTORS* | “FORCE” | “COORDINATION” | “RW PERFORMANCE” | “DISEASE” |
| Genotype | F=32.72, *p<0.0001* | F=0.00, p=0.98 | F=6.62, *p=0.01* | --- |
| Sex | F=39.33, *p<0.0001* | F=32.27, *p<0.0001* | F=33.38, *p<0.0001* | F=0.75, p=0.39 |
| Exercise Group | F=17.67, *p<0.0001* | F=28.23, *p<0.0001* | F=54.75, *p<0.0001* | F=3.98, *p=0.02* |
| Genotype*Sex | F=0.71, p=0.40 | F=1.39, p=0.24 | F=0.00, p=0.97 | --- |
| Genotype*Exercise Group | F=0.97, p=0.38 | F=0.10, p=0.90 | F=0.70, p=0.40 | --- |
| Sex*Exercise Group | F=10.65, *p<0.0001* | F=2.96, p=0.056 | F=0.35, p=0.55 | F=6.36, *p=0.003* |

ANOVA Table summarizing the results (F and *p*-values) obtained from the most relevant components extracted from the Principal Components Analysis (PCA) on 1) all mice (n=108), FORCE and COORDINATION; 2) all running mice (n=75), RW PERFORMANCE; 3) only mutant mice (n=52), DISEASE. Statistically significant factor’s *p*-values are in *italics*.
